# Ship Rolling Bearing Fault Identification Under Complex Operating Conditions: Multi-Domain Feature Extraction-Based LCM-HO Enhanced LSSVM Approach

**DOI:** 10.3390/s25175400

**Published:** 2025-09-01

**Authors:** Qiang Yuan, Jinzhi Peng, Xiaofei Wen, Zhihong Liu, Ruiping Zhou, Jun Ye

**Affiliations:** 1 School of Naval Architecture and Maritime, Zhejiang Ocean University, Zhoushan 316022, China; pengjinzhi@zjou.edu.cn (J.P.); liuzhihong_only@163.com (Z.L.); 2 School of Naval Architecture, Ocean and Energy Power Engineering, Wuhan University of Technology, Wuhan 430070, China; rpzhou@whut.edu.cn (R.Z.); j.ye@whut.edu.cn (J.Y.)

**Keywords:** ship bearings, rolling bearings, fault diagnosis, least squares support vector machine, hippo algorithm, chaotic mapping

## Abstract

With the continuous advancement of intelligent, integrated, and sophisticated modern marine equipment, bearing fault diagnosis faces increasingly severe technical challenges. Compared with traditional industrial environments, marine propulsion systems are characterized by multi-bearing coupled vibrations and complex operating conditions. To address these characteristics, this paper proposes a fault diagnosis method that combines a least squares support vector machine (LSSVM) with multi-domain feature extraction based on an improved hippopotamus optimization algorithm (LCM-HO). This method directly extracts time, spectral, and time-frequency domain features from the raw signal, effectively avoiding complex preprocessing and enhancing its potential for field engineering applications. Experimental verification using the Paderborn bearing dataset and a self-built marine bearing test bench demonstrates that the LCM-HO-LSSVM method achieves diagnostic accuracy rates of 99.11% and 98.00%, respectively, demonstrating significant performance improvements. This research provides a reliable, efficient, and robust technical solution for bearing fault diagnosis in complex marine environments.

## 1. Introduction

With the rapid development of intelligent shipping technology, the shipbuilding industry is undergoing a profound transformation from traditional manufacturing to intelligent and digitalized production. To guide this change, major shipping nations and international organizations have successively introduced relevant regulations. For instance, the China Classification Society (CCS) has issued the “Smart Ship Specifications,” and the International Association of Classification Societies (IACS) has also successively promulgated corresponding specifications and standards [1]. In this context, equipment health management has become one of the key supporting technologies for smart ships. As a core component of the shaft system, rolling bearings have a high failure rate, and their health status is directly related to the operational safety of the entire shaft system. Should a failure occur, it may cause irreversible damage to the vessel, leading to serious maritime safety incidents and significant economic losses [2,3,4]. Therefore, research on the health condition monitoring of rolling bearings is not only of great theoretical value but also holds significant practical importance for ensuring vessel safety and enhancing its intelligence level.

However, the vibration signals of rolling bearings exhibit complex characteristics under the influence of factors such as high-speed vessel operation, multi-source noise interference, and multi-fault coupling, leading to challenges like difficult feature extraction and low fault diagnosis accuracy [5]. The multi-bearing coupled configuration within marine propulsion systems means that fault signals not only contain their own features but are also affected by the coupled dynamics of adjacent bearings and the shaft system structure, which further exacerbates feature masking and recognition difficulty. At the same time, the deployment of on-site detection equipment is subject to practical constraints such as spatial limitations, harsh environmental conditions, and real-time requirements, making it difficult for traditional diagnostic methods to be directly applied in marine engineering practice.

In the current field of rolling bearing fault diagnosis, extensive research has been conducted on vibration signal processing and feature extraction. Traditional methods primarily focus on single analysis domains, including various signal processing techniques like time-domain, frequency-domain, and time-frequency analysis. Time-domain analysis reflects the overall characteristics of a signal through statistical parameters such as the root mean square value and peak factor; frequency-domain analysis reveals periodic components; and time-frequency analysis combines both time and frequency information, making it suitable for processing non-stationary signals [6]. With the advancement of signal processing technology, methods such as short-time Fourier transform (STFT) [7], wavelet transform (WT) [8], empirical mode decomposition (EMD) [9], and variational mode decomposition (VMD) [10] have further enhanced the effectiveness and accuracy of feature extraction. Furthermore, research into enhancing signal processing performance and feature extraction is continuously deepening, with new techniques like VMD parameter optimization [11], blind deconvolution based on candidate fault frequencies [12], and envelope spectrum optimization methods [13] providing effective support for the extraction and identification of fault signals.

However, studies have shown that single-domain signal processing methods still suffer from incomplete information extraction and poor generalization capabilities under complex operating conditions. Consequently, multi-domain feature fusion methods have emerged as a research hotspot in recent years. By integrating signal features from different analysis domains, these approaches can more comprehensively capture fault characteristics and significantly enhance diagnostic accuracy and robustness. Several notable contributions have advanced this field. Yan et al. [14] proposed an SVM classification algorithm with multi-domain feature optimization for bearing fault diagnosis. Wang et al. [15] developed the MDF-Relief-Bayes-KNN model, which integrates Relief feature selection with Bayesian optimization for k-nearest neighbors classification. Li et al. [16] presented a multi-level feature fusion strategy for multi-domain vibration signal analysis. Additionally, Wang et al. [17] introduced a fault diagnosis method that combines multi-domain features with whale optimization support vector machines. These studies have demonstrated the effectiveness of multi-domain approaches in improving diagnostic performance.

While multi-domain feature extraction has shown significant promise, effectively utilizing these extracted features for accurate fault classification presents another critical challenge. Pattern recognition, as the core component of fault diagnosis systems, directly determines classification performance and substantially influences overall diagnostic accuracy. Traditional machine learning methods have been extensively applied in bearing fault diagnosis, including classification and regression trees (CARTs) [18], random forests (RFs) [19,20], the AdaBoost algorithm [21], support vector machines (SVMs) [22], and k-nearest neighbor classification (K-NN) [23]. In recent years, deep learning approaches have gained considerable attention in this domain. Deep learning-based models, such as convolutional neural networks (CNNs) [24], recurrent neural networks (RNNs) [25], and long short-term memory networks (LSTMs) [26], demonstrate superior capability in automatically extracting high-level features and exhibit exceptional performance in large-scale data environments. However, these methods typically require substantial amounts of labeled training data and suffer from limited model interpretability.

Although each of the aforementioned methods has its advantages, the complexity of marine equipment and the coupled characteristics of bearing failures pose higher demands on algorithm performance [27,28,29]. Support vector machines (SVMs) enhance model generalization capabilities by minimizing structural risk; however, they still face limitations including high computational complexity, parameter sensitivity, and insufficient performance with small sample sizes [30]. The least squares support vector machine (LSSVM), an improved version of the SVM algorithm, replaces inequality constraints with linear least squares, combining strong generalization capabilities with low computational costs, making it particularly suitable for the real-time engineering requirements of ships. Gao et al. [31] proposed a rolling bearing fault diagnosis method based on LSSVMs, which achieved high diagnostic performance within a brief classification period.

However, the diagnostic performance of LSSVMs heavily relies on the correct selection of penalty parameters and kernel function parameters. Manual parameter tuning is not only time-consuming but also fails to achieve optimal results under varying operating conditions. To address this issue, parameter optimization algorithms are necessary for automatic optimization. Deng et al. [32] proposed an enhanced particle swarm optimization (PSO) algorithm to optimize LSSVM parameters, thereby constructing an optimal LSSVM classifier for fault classification. Chen et al. [33] introduced a bearing fault diagnosis method that integrates an improved grey wolf optimization (IGWO) algorithm with the LSSVM model. Furthermore, Jin et al. [34] suggested a fault diagnosis technique based on a modified particle swarm optimization (MPSO) algorithm to enhance the LSSVM fault diagnosis method. Research indicates that swarm intelligence optimization algorithms represent a viable approach to improving diagnostic efficiency and performance.

Recently, numerous scholars have introduced various innovative swarm intelligence algorithms. Among these, the hippopotamus optimization algorithm (HO) was proposed by Amiri et al. [35] in 2024. This algorithm is inspired by the behavioral patterns of hippopotamuses, simulating their positional updates, defense strategies, and evasion methods in rivers or ponds. The HO algorithm is notable for its exceptional performance, enabling it to rapidly identify and converge towards the optimal solution while effectively avoiding local minima. However, it still remains susceptible to performance degradation and the occurrence of local optima when faced with complex problems [36].

This paper addresses two key challenges in rolling bearing fault diagnosis: (1) using raw signal feature vectors for multi-domain feature extraction to comprehensively evaluate bearing health status, and (2) developing a high-precision LSSVM fault identification model with adaptive parameter optimization. The main contributions of this work are as follows:

(1) Time-frequency joint analysis is utilized for multi-domain feature extraction, comprehensively characterizing the bearing’s health status through statistical indices such as vibration energy and spectral characteristics.
(2) A novel hippopotamus optimization algorithm (LCM-HO) improved with chaos mapping is proposed. By enhancing population diversity, this algorithm effectively strengthens the global optimization and local optima escape capabilities of the meta-heuristic algorithm. The LCM-HO algorithm is then applied to adaptively optimize the kernel parameters and penalty factors of the least squares support vector machine (LCM-HO-LSSVM).
(3) The superiority of the LCM-HO algorithm is verified through multi-metric comparisons with HO, GWO, and SSA using test functions. Furthermore, the proposed LCM-HO-LSSVM intelligent diagnostic method is validated using the University of Paderborn (PU) bearing dataset and a self-built marine bearing test platform. Evaluation results based on confusion matrices and accuracy rates confirm its superior diagnostic precision and generalization performance.

The structure of this paper is organized as follows: Section 2 introduces the fundamental principles of HO-LSSVM. Section 3 describes the LCM-HO algorithm and its LSSVM parameter optimization process. Section 4 presents the proposed fault diagnosis framework. Section 5 validates the effectiveness of the proposed method using the PU dataset and self-collected experimental data, comparing its performance, feature extraction, and fault identification effects. Section 6 provides conclusion and future works.

## 2. HO-LSSVM Principles and Methods

### 2.1. LSSVM

LSSVM [37] represents an enhanced variant of the SVM methodology, designed to identify optimal functions for data fitting through squared error sum minimization, as demonstrated in Equation (1), offering superior generalization capabilities with reduced computational expenses.

(1) $$\min\sum_{i=1}^{n}e_{i}^{2}=\min\sum_{i=1}^{n}{\left(Y_{i}-\hat{Y_{i}}\right)}^{2}$$

where, $e_{i}$ is the error term, $Y_{i}$ is the predicted value, and $\hat{Y_{i}}$ is the actual value.

Within the LSSVM framework, error terms undergo dual-paradigm processing, constraint inequalities are converted to equality expressions, squared errors are incorporated into the objective function, and optimization is achieved through Karush–Kuhn–Tucker conditions (KKT). This approach effectively minimizes problem-solving complexity while decreasing computational requirements. The LSSVM optimization function is presented in Equation (2).

(2) $$\min J\left(\omega ,b,e\right)\frac{1}{2}{∥\omega ∥}^{2}+\frac{\gamma }{2}\sum_{i=1}^{n}e_{i}^{2}$$

$$\begin{array}{cc} s.t.\; & y_{i}\left(\varphi \left(x_{i}\right)\omega^{T}+b\right)=e_{i}+1\\ \; & i=1,2,\ldots ,n \end{array}$$

where ω is the weight vector and γ is the penalty factor.

The Lagrangian formula is as follows in Equation (3):

(3) $$L\left(\omega ,b,e,a\right)=\frac{1}{2}{∥\omega ∥}^{2}+\frac{\gamma }{2}\sum_{i=1}^{n}e_{i}-\sum_{i=1}^{n}a_{i}\left(y_{i}\left(\omega^{T}\varphi \left(x_{i}\right)+b\right)-1+e_{i}\right)$$

where *b* represents the bias parameter, *a* denotes the Lagrange multiplier, and $a_{i}$ constitutes the multiplier vector.

Computing the partial derivatives of ω, *b*, *a*, *e*, setting them equal to zero enables parameter determination through linear system solution. Furthermore, the classification decision function is derived as

(4) $$f\left(x\right)=sign\left(\sum_{i=1}^{n}a_{i}y_{i}K\left(x,x_{i}\right)+b\right)$$

where $K\left(x,x_{i}\right)$ represents the kernel function, employed for transforming input vectors from lower-dimensional spaces into higher-dimensional representations.

Kernel functions encompass various types including polynomial functions, radial basis functions, and Sigmoid functions. This research methodology employs the RBF kernel function.

### 2.2. Principle of HO Algorithm

The HO algorithm represents a metaheuristic approach inspired by hippo herd behaviors, encompassing four essential phases:

(1) Initialization Phase:

The algorithm treats hippo individuals as candidate solutions, establishing an initial population matrix through random generation strategies. Each hippo’s position vector represents decision variable values, with population size n and decision variable dimension m. Position initialization follows uniform distribution principles:

(5) $$x_{i}:x_{ij}=lb_{j}+r\cdot \left(ub_{j}-lb_{j}\right),i=1,2,\ldots ,n;j=1,2,\ldots ,m$$

(6) $$x={\left[\begin{array}{c} x_{1}\\ \vdots \\ x_{i}\\ \vdots \\ xN \end{array}\right]}_{n\times m}={\left[\begin{array}{ccccc} x_{1,1} & \ldots & x_{1,j} & \ldots & x_{1,m}\\ \vdots & \ddots & \vdots & ⋰ & \vdots \\ x_{i.1} & \ldots & x_{i,j} & \ldots & x_{i,m}\\ \vdots & ⋰ & \vdots & \ddots & \vdots \\ x_{n,1} & \ldots & x_{n,j} & \ldots & x_{n,m} \end{array}\right]}_{n\times m}$$

where $x_{i}$ denotes the location of the *i* th candidate solution, *r* represents a random number within [0-1], and lb and ub indicate the boundary values for the *j*-th decision variable.

(2) Exploration Phase—Position Update Mechanism:

The hippo social structure comprises adult females, calves, multiple adult males, and a dominant male. The dominant individual is dynamically determined based on objective function values. The position update strategy for male hippos is as follows:

(7) $$\begin{array}{c} x_{i}^{Mhippo}:x_{ij}^{Mhippo}=x_{ij}+y1\cdot \left(Dhippo-I_{1x_{ij}}\right)\\ fori=1,2,\ldots ,\left[\frac{N}{2}\right]andj=1,2,\ldots ,m \end{array}$$

where $x_{i}^{Mhippo}$ indicates the position of the male hippopotamus and Dhippo indicates the position of the dominant hippopotamus. This mechanism simulates the hierarchical system of hippo herds, guiding search directions through dominant individuals.

(3) Defense Behavior Phase:

The primary purpose of hippo herding is security provision. When facing external threats, the herd employs collective defense strategies. The mathematical model reflects hippo responses to predator encounters:

(8) $$\begin{array}{c} x_{i}^{HippoE}:x_{ij}^{HippoE}=\left\{\begin{array}{c} \vec{RL}⊕\mathrm{Pr}edator_{j}+\left(\frac{f}{\left(c-d\times \cos(2\pi g\right)}\right)\cdot \left(\frac{1}{\vec{D}}\right)F\mathrm{Pr}edator_{j}<F_{i}\\ \vec{RL}⊕\mathrm{Pr}edator_{j}+\left(\frac{f}{\left(c-d\times \cos(2\pi g\right)}\right)\cdot \left(\frac{1}{2\times \vec{D}+\vec{r_{9}}}\right)F\mathrm{Pr}edator\geq F_{i} \end{array}\right.\\ fori=\left[\frac{N}{2}\right]+1,\left[\frac{N}{2}\right]+2,\ldots ,Nandj=1,2,\ldots ,m \end{array}$$

where $x_{i}^{HippoP}$ is the position of the predator.

(4) Escape Behavior Phase:

When defense strategies prove ineffective or when confronting overwhelming threats, individual hippos seek safer territories. This phase simulates the algorithm’s local search capabilities:

(9) $$\begin{array}{c} x_{i}^{HippoE}:x_{ij}^{HippoE}=x_{ij}+\vec{r_{10}}\cdot \left(lb_{j}^{local}+s_{1}\cdot \left(ul_{j}^{local}-lb_{j}^{local}\right)\right)\\ i=1,2,\ldots ,N,j=1,2,\ldots m \end{array}$$

where $x_{i}^{HippoE}$ is the location of the hippopotamus that is searching for a safe place. This phase enhances the algorithm’s local exploitation ability, facilitating precise optimal solution identification.

### 2.3. HO-LSSVM Framework

LSSVM is widely utilized in fault diagnosis due to its remarkable classification performance, reliability, and speed. However, its performance can be significantly influenced by the penalty factor and kernel parameters, which represent the two dimensions of an individual. The HO algorithm offers advantages such as straightforward parameters, robust search capabilities, rapid convergence, and ease of implementation, making it suitable for hyperparameter selection in LSSVM to enhance classification accuracy. In this study, HO is employed to optimize the two critical parameters, σ2 (sig2) and γ (gam) of LSSVM, to improve the accuracy of bearing diagnosis. The optimization process involves the following steps:

Step 1: Define the population size, maximum number of iterations, search space dimension, and HO parameters, followed by the random generation of hippopotamus locations within the specified range.

Step 2: Establish the parameter range and initialize the LSSVM settings.

Step 3: Compute the fitness function value of the HO at the current location, continuously updating both the location and fitness function value according to Equations (7) and (8).

Step 4: Assess the number of iterations; if it does not exceed the maximum limit, return to Step 3. Otherwise, the optimization concludes, and the optimal parameters are output.

Step 5: Train the LSSVM using the optimal parameters and apply the trained model to classify faults within the test set.

## 3. LCM-HO-LSSVM Principles and Methods

### 3.1. Principle of LCM-HO Algorithm

**Figure 1:**
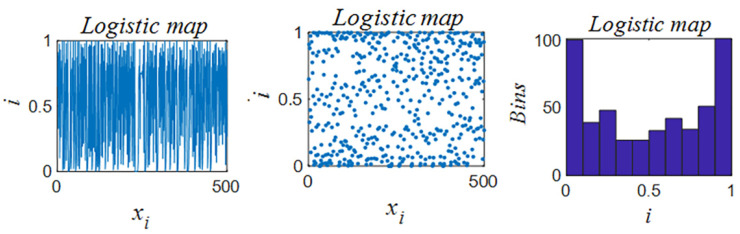
Logistic chaos mapping distribution.

The operational effectiveness of ship rolling bearings is influenced by numerous factors, including the intricate design of the machinery, diverse operational conditions, and the prevailing vibration environment. These factors contribute to the likelihood of multi-location, multi-degree, and multi-type compound faults. To address the issue that HO algorithms are susceptible to local optimization in complex fault scenarios [38], this study proposes the implementation of logistic chaos mapping to enhance population diversity. this mapping generates pseudo-random behaviors in deterministic systems through nonlinear properties, and its sensitivity to initial conditions and parameter-modulated chaotic characteristics can continuously perturb the search process, thereby avoiding premature convergence. Figure 1 shows the distribution of the chaotic mapping. The efficacy of this method has been demonstrated in enhancing the algorithm’s capacity to identify composite faults and suppressing the impact of environmental noise while ensuring computational efficiency.

The principle equation is shown in Equation (10):

(10) $$x_{ij}^{k+1}=\mu x_{ij}^{k}\left(1-x_{ij}^{k}\right)$$

where μ is the parameter that determines the behavior of the mapping and $x_{ij}^{k+1}$ is a chaotic mapping of $x_{ij}^{k}$.

The detailed implementation steps of the LCM-HO algorithm are as follows:

(1) Execute the logistic chaos mapping algorithm to initialize the hippopotamus population;
(2) Initialize the HO parameters, optimize the parameter range, and generate random positions;
(3) Update the best-adapted individual of the hippopotamus based on the calculation results corresponding to different combinations of parameters;
(4) Update the position of the hippopotamus based on the chaotic mapping strategy;
(5) Return to step 3 for iterative looping until the maximum number of iterations is reached and the optimal parameter combination is output.

**Figure 2:**
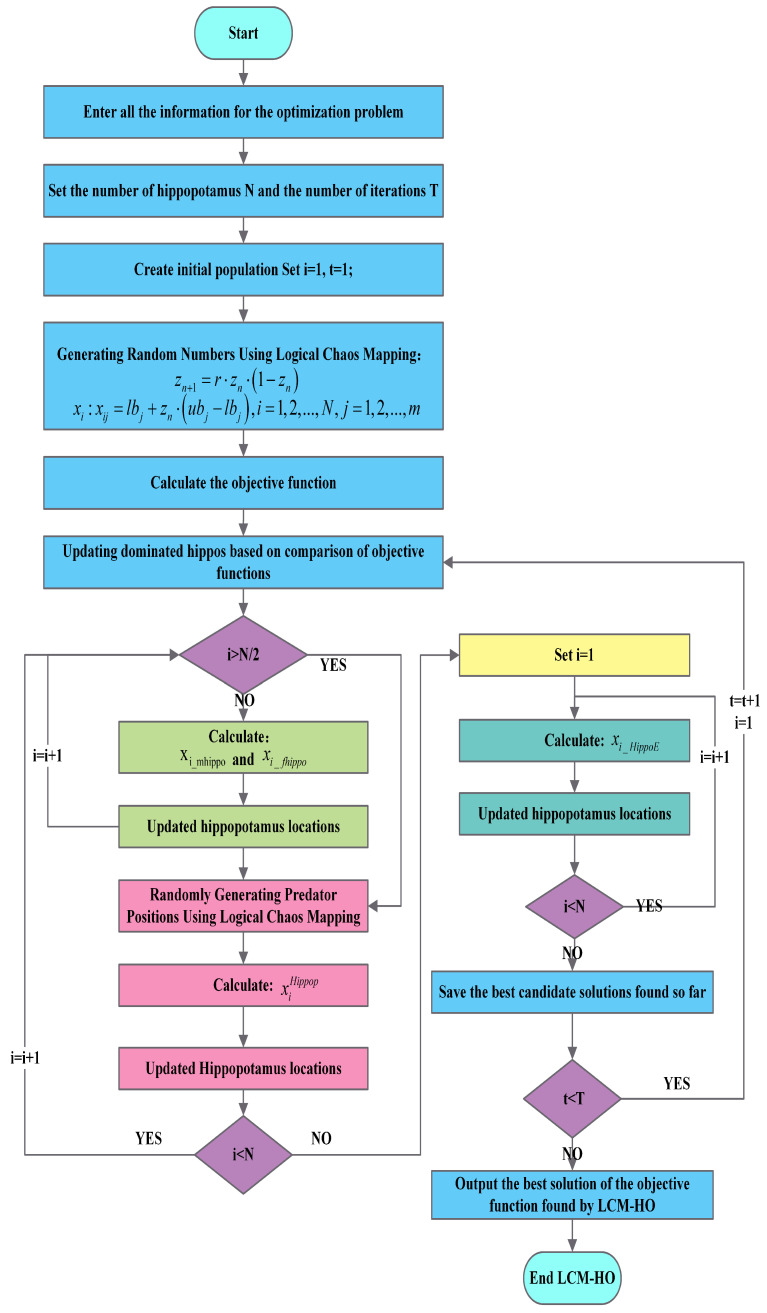
Flowchart of the LCM-HO algorithm.

The flowchart is shown in Figure 2.

### 3.2. LCM-HO-LSSVM Framework

**Figure 3:**
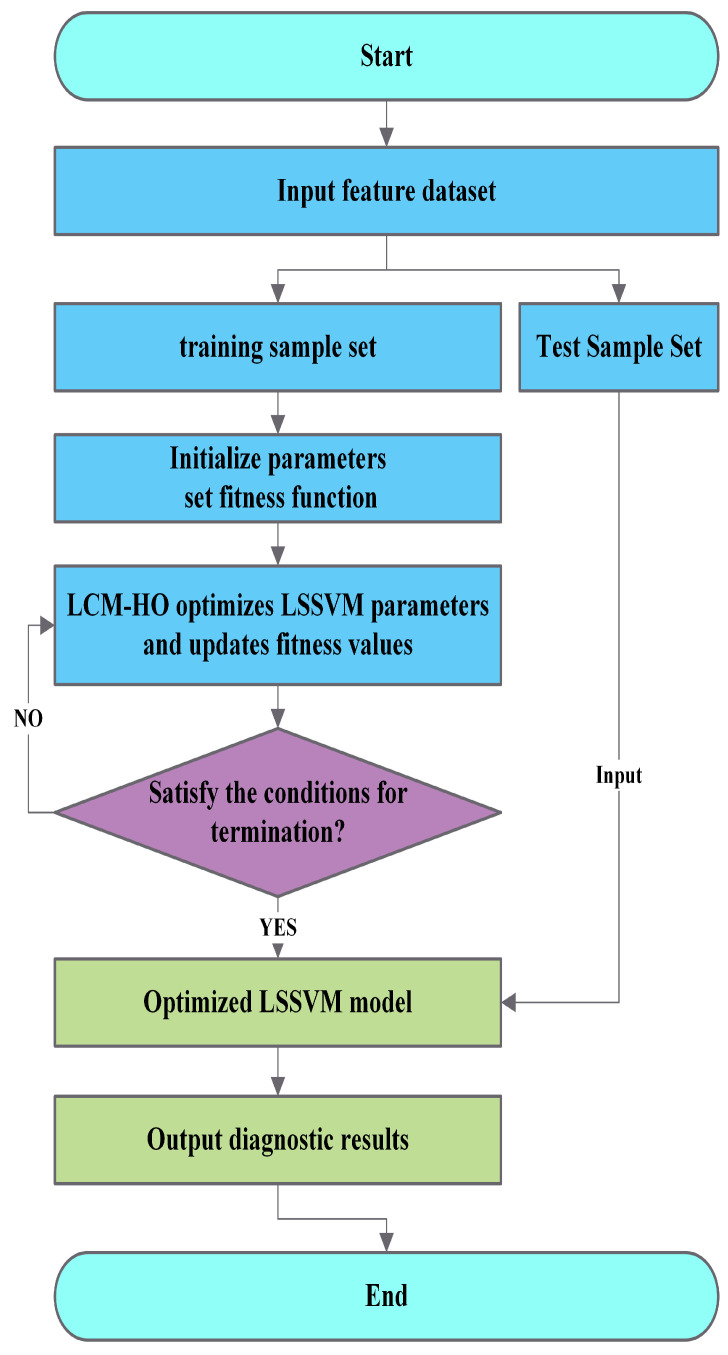
Flowchart of the LCM-HO-LSSVM.

The LCM-HO algorithm is used to optimize the two key parameters (σ2 and γ) of the LSSVM to improve the accuracy of bearing diagnosis. Figure 3 shows the specific flowchart. The exact operation stages are the same as above, and the LCM-HO method can be used in place of HO.

## 4. Fault Diagnosis Framework

### 4.1. Multi-Domain Feature Extraction

The construction of a multi-domain feature fusion diagnostic system involves the utilization of a time domain–frequency domain–time-frequency domain joint analysis method. This method is employed to establish a multi-dimensional feature space, thereby facilitating the formation of a complementary feature characterization system. The time-domain feature group focuses on the statistical characteristics of the signal waveform, the frequency-domain feature group analyses the frequency components of periodic faults, and the time-frequency domain feature group captures the transient impact response. The synergy of these three groups significantly improves fault identification accuracy under complex working conditions.

The time-domain feature group emphasizes the statistical characteristics of the signal waveform, the frequency-domain feature group analyzes the frequency components of periodic faults, and the time-frequency domain feature group captures transient impact responses. The synergy of these three groups significantly enhances fault identification accuracy under complex working conditions. The time-domain feature comprises three constituent components, root mean square (RMS), variance (Var), and peak-to-peak (PP), which collectively characterize the energy level and fluctuation intensity of the signal. These parameters are employed to assess the overall operational status of the equipment. The peak value (PvT), kurtosis indicator (K), and impulse indicator (I) are susceptible to impact faults. Consequently, these indicators can detect abnormalities in rotating components, such as bearings, at an early stage. Additionally, the margin indicator (L), line integral (LI), peak indicator (C), waveform indicator (W), and skewness indicator (S) characterize the signal waveform from various perspectives, facilitating the differentiation of different fault patterns. The above characteristic formulas are shown in Table 1.

Frequency domain features reveal the frequency composition of signals, with the spectral peak value (PvF) and spectral energy (En) effectively identifying changes in characteristic frequency and energy distribution associated with specific faults. This provides a diagnostic basis for mechanical component faults, such as unbalance and misalignment. Time-frequency domain features, which combine time and frequency information, utilize wavelet packet decomposition to analyze the energy characteristics of the eight decomposition signals. This approach can accurately locate fault distributions in the time-frequency plane, making it particularly suitable for analyzing the transient characteristics of non-smooth signals. The above characteristic formulas are shown in Table 2.

The construction of a multi-domain feature combination model enhances the accurate identification and classification of various fault types in complex systems. This improvement arises from its ability to overcome the limitations of single features, which often fail to represent fault states comprehensively. Additionally, the model increases diagnostic accuracy and reliability, offers diverse feature inputs for intelligent fault identification, and effectively addresses the recognition challenges posed by coupled feature signals in complex equipment systems.

### 4.2. Fault Diagnosis Model

**Figure 4:**
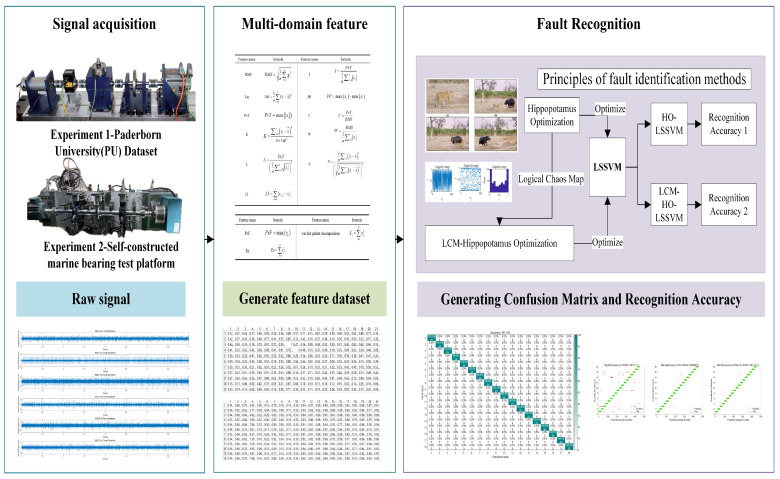
Flow chart of rolling bearing fault diagnosis.

To achieve effective rolling bearing fault diagnosis, a multidomain feature dataset comprising 21 feature indicators across the time domain, frequency domain, and time-frequency domain is derived from the original vibration signals. This dataset is subsequently inputted into a parameter-optimized LSSVM classifier for fault pattern recognition. Figure 4 illustrates the flowchart of the defect diagnostic technique based on the parameter-optimized LSSVM and multi-feature extraction. The steps are as follows:

Step 1: Identify k fault types in the bearing signal dataset and collect i sets of samples for each type;

Step 2: Calculate the multi-domain features for each signal type as input feature vectors;

Step 3: Construct a new feature vector set from the optimized features, assign labels according to the fault types, select a portion of the samples from each type as training samples, and reserve the remaining samples as test samples, thereby establishing the training sample feature set and the test sample feature set, respectively;

Step 4: Train the parameter-optimized LSSVM using the training set while automatically selecting the optimal parameter combination based on the HO algorithm and LCM-HO algorithm;

Step 5: Conduct fault identification regarding fault types and degrees for the test samples utilizing the trained HO-LSSVM and LCM-HO-LSSVM.

## 5. Experiment

### 5.1. Algorithm Performance Test

To verify the performance advantages of the LCM-HO algorithm, this paper refers to the test functions mentioned in reference [39]. A comparative analysis is conducted with the original HO algorithm, GWO, and SSA across multiple dimensions. Table 3 provides a concise overview of the mathematical expressions, search ranges, and theoretical optimal values for the seven benchmark test functions. The functions F1–F4 are unimodal and designed to assess convergence accuracy and depth search capability. In contrast, functions F5–F7 are multimodal, facilitating the evaluation of the algorithm’s ability to address complex problems and the efficacy of global optimization searches.

This study refers to the multi-strategy metaheuristic optimization algorithm performance evaluation methods widely used in references [40,41], and quantitatively evaluates algorithm performance through four core indicators. (1) Min: characterizes the algorithm’s best optimization ability across multiple runs. (2) Avg: reflects the comprehensive performance of the algorithm in escaping local optimal solutions, with values closer to the theoretical optimal indicating better optimization. (3) Std: measures the degree of performance variability, tending towards 0 when demonstrating strong robustness. (4) Max: reveals the risk boundary of the algorithm and its engineering applicability. Each index constructs a complete evaluation system from four dimensions: ideal performance, average performance, stability threshold, and risk control.

To ensure the fairness of the experiment, all algorithms must utilize the same settings. To mitigate random errors, parameters are set to *N* = 30, dim = 30, and *T* = 400, and each algorithm is executed independently 30 times. The Min, Avg, Std, and Max computation outcomes for the four methods are detailed for each of the seven test functions, as shown in Table 4. This provides a solid foundation for the subsequent analysis.

The LCM-HO algorithm demonstrates significant advantages across all evaluated functions, as evidenced by the analysis of the experimental data. This method achieves the theoretically optimal value of 0 for the F1 (unimodal), F6 (multimodal), and F7 (multimodal) functions, as illustrated in Table 4. Despite the F2–F4 functions not achieving full convergence, their convergence accuracy (Min) is, notably, 0.5 to 2 orders of magnitude superior to that of the HO algorithm, 0.5 to 22 orders of magnitude better than the SSA algorithm, and 2 to 152 orders of magnitude superior to the GWO technique. Remarkably, in complex multi-peak function scenarios such as F5–F6, the LCM-HO approach retains its accuracy advantage while achieving convergence outcomes comparable to those of the HO and SSA algorithms.

In terms of stability, the LCM-HO algorithm excels in two critical indices: standard deviation and mean value. The standard deviation and mean value across all test functions are maintained at their lowest levels, with the standard deviation for F1 and F5–F7 stabilized at 0, which substantiates its robustness. Furthermore, from the perspective of risk prevention and control, the worst-case value of LCM-HO is improved when compared to the next-best algorithms, indicating an enhancement in its risk control capabilities.

In conclusion, logistic chaos mapping significantly enhances the HO algorithm, facilitating an optimization process characterized by both high precision convergence and strong resilience against interference. With the optimization algorithm validated, the following experiments evaluate the complete LCM-HO-LSSVM framework for bearing fault diagnosis using two datasets: the Paderborn University dataset for general validation and a self-constructed ship bearing test rig for marine-specific applications.

### 5.2. Experiment 1—PU Dataset

**Figure 5:**
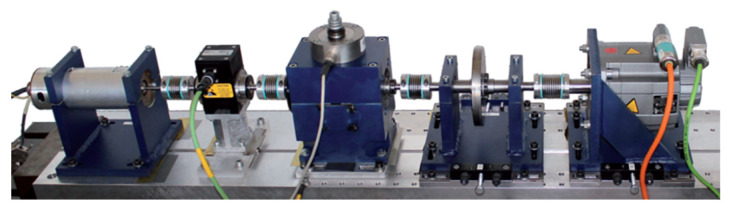
Test bed for data testing.

**Figure 6:**
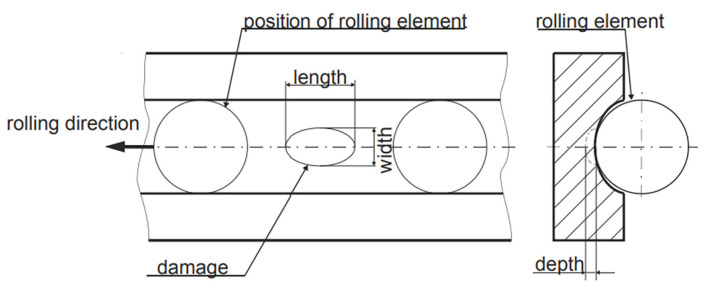
Damage geometry.

The bearing dataset from the University of Paderborn in Germany features a wide range of operating conditions, and the signals collected from its accelerated life test rig are more complex than those from artificial damage signals, making them more representative of realistic bearing fault characteristics. As a result, using this dataset can better validate the effectiveness of this method. This benchmark dataset, established by Lessmeier et al. [42], is derived from the 6203 deep groove ball bearing test rig (Figure 5), covering three failure modes, single-point damage on the inner/outer rings, composite damage (including pitting, corrosion, and indentations caused by both damage mechanisms), with damage severity categorized into three levels (Figure 6, Table 5).

**Figure 7:**
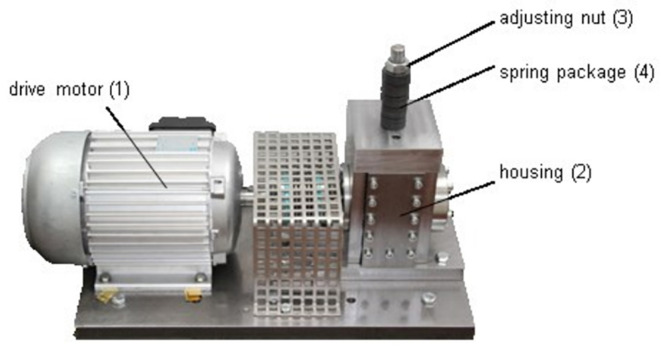
Accelerated life test rig.

The specific data collection settings are as follows:

(1) Data acquisition: obtained via an accelerated life test stand (Figure 7). The types and severity of bearing damage are detailed in Table 6 and Table 7.
(2) Operating conditions: Four load-speed combination conditions were set (Table 8).
(3) Sampling parameters: The vibration signal sampling frequency is 64 kHz, with a single sampling duration of 4 s.
(4) Data processing: Each sample was extracted with 4096 data points to balance computational efficiency and analytical accuracy.

Based on the above settings, 20 valid samples were collected for each damage type under the four operating conditions, ultimately constructing a multidimensional comprehensive dataset encompassing failure mechanisms, damage locations, and damage severity.

**Table 5:** Determination of impairment rating by degree of impairment [42].

Damage Level	Specify Percentage Value	6203 Bearing Limit
1	0–2%	≤2 mm
2	2–5%	>2 mm
3	5–15%	<4.5 mm

**Table 6:** Classification of healthy bearing-related information and labeling [42].

Bearing Status	Number	Runtime/h	Radial Force	Speed	Sample Size	Label
Health	K001	50	1000–3000	1500–2000	80	1
	K002	19	3000	2900	80	2
	K003	1	3000	3000	80	3
	K004	5	3000	3000	80	4
	K005	10	3000	3000	80	5

**Table 7:** Damage bearing failure types and label classification [42].

Bearing Status	Number	Injury Symptoms	Damaged Package	Injury Programming	Damage Level	Damage Characteristics	Sample Size	Label
Outer ring (OR)	KA04	Fatigue Pitting	S	No Repeat	1	Single point	80	6
	KA15	Indentation	S	No Repeat	1	Single point	80	7
	KA16	Fatigue Pitting	R	Random	2	Single point	80	8
	KA22	Fatigue Pitting	S	No Repeat	1	Single point	80	9
	KA30	Indentation	R	Random	1	Distributed	80	10
Inner ring (IR)	KI04	Fatigue Pitting	M	No Repeat	1	Single point	80	11
	KI14	Fatigue Pitting	M	No Repeat	1	Single point	80	12
	KI16	Fatigue Pitting	S	No Repeat	3	Single point	80	13
	KI18	Fatigue Pitting	S	No Repeat	2	Single point	80	14
	KI21	Fatigue Pitting	S	No Repeat	1	Single point	80	15
Composite damage (C)	KB23	Fatigue Pitting	M	Random	2	Single point	80	16
	KB24	Fatigue Pitting	M	No Repeat	3	Distributed	80	17
	KB27	Indentation	M	Random	1	Distributed	80	18

Note: In damage combination, S: bearing single component produces one type of damage; R: bearing single component multiple same type of damage; M: different type of damage/same type of damage on different components.

**Table 8:** Four working conditions [42].

No.	Rotation Speed/Rpm	Load Torque/Nm	Radial Force/N	Setting Name
1	1500	0.7	1000	N15_M07_F10
2	900	0.7	1000	N09_M07_F10
3	1500	0.1	1000	N15_M01_F10
4	1500	0.7	400	N15_M07_F04

#### 5.2.1. Fault Identification Results 1

All computational experiments were conducted on a computer with the following specifications: 13th Gen Intel(R) Core(TM) i9-13900H processor (Intel Corporation, Santa Clara, CA, USA) @ 2.60 GHz, 32.0 GB RAM (Micron Technology, Boise, ID, USA), Windows 11 operating system, and MATLAB R2023a environment.

The raw signals collected from the experimental test rig were processed to extract multi-domain features, resulting in several feature datasets, as illustrated in Table 9. These obtained feature datasets were subsequently imported into the classifiers: LSSVM, HO-LSSVM, and LCM-HO-LSSVM. Notably, the initial parameters for HO-LSSVM and LCM-HO-LSSVM were set as follows: The number of hippopotamus populations was 20, the maximum number of iterations was 30, and the search parameter range was defined with Lowerbound = [$1\times 10^{-4}$, $1\times 10^{-4}$] and Upperbound = [$1\times 10^{4}$, $1\times 10^{4}$]. The values for gam and sig2 were randomly assigned as gam = 1.2 and sig2 = 1.5.

**Figure 8:**
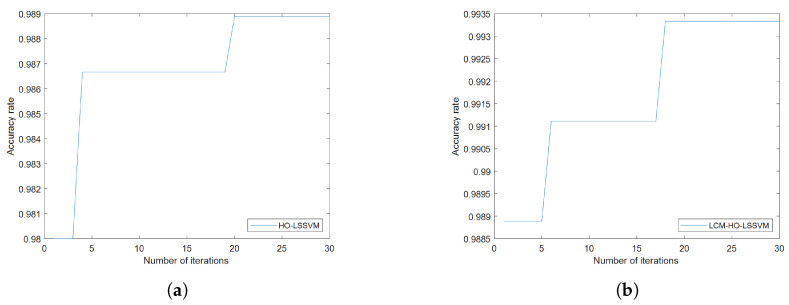
Convergence curves of HO-LSSVM and LCM-HO-LSSVM after 30 iterations. (**a**) HOLSSVM iteration curve; (**b**) LCM-HO-LSSVM iteration curve.

**Figure 9:**
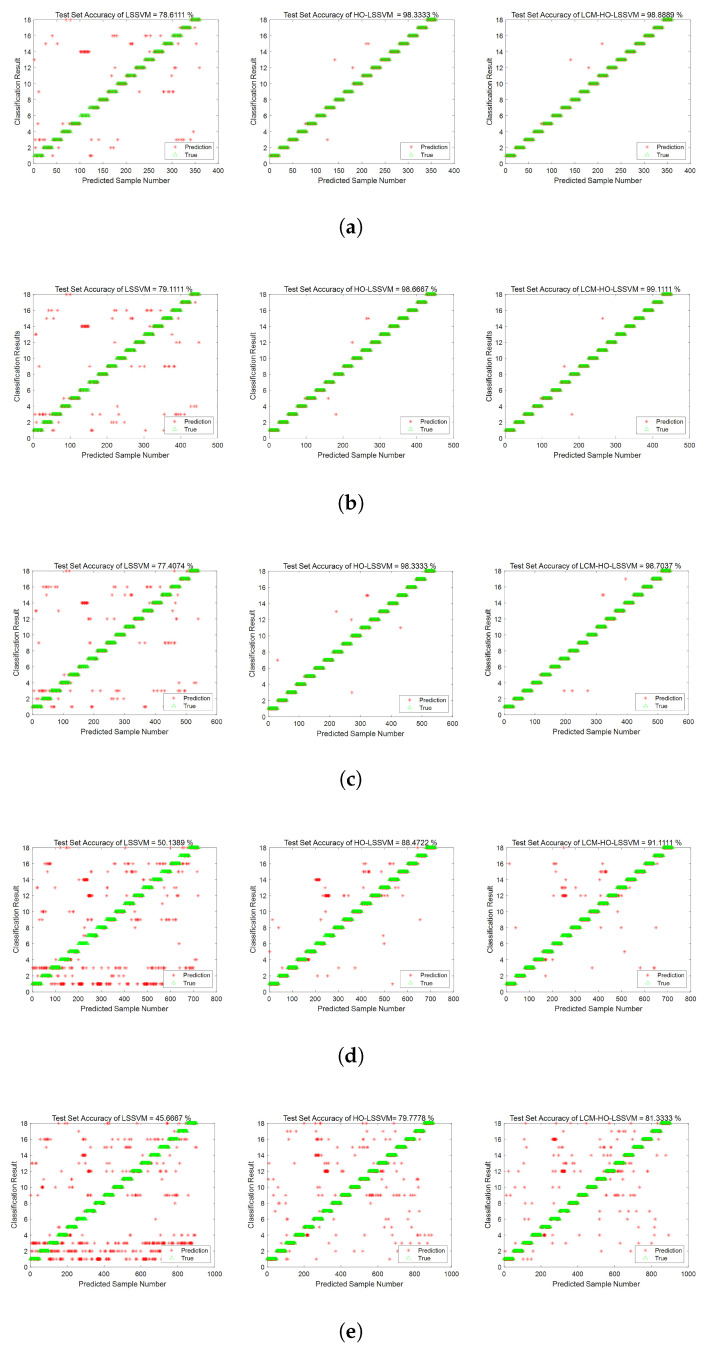
Accuracy rate of three classifiers at different ratios. (**a**) Ratio 1. (**b**) Ratio 2. (**c**) Ratio 3. (**d**) Ratio 4. (**e**) Ratio 5.

**Figure 10:**
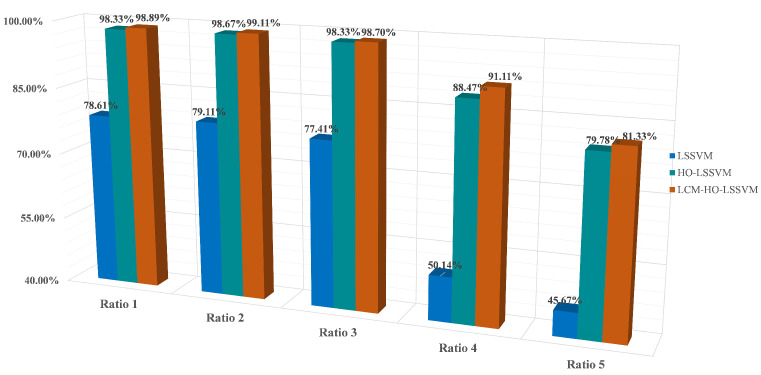
Accuracy of different proportions for each classifier.

Furthermore, to assess the classifier’s performance, experiments were conducted on training and test samples with varying ratios. During the fault diagnosis process under ratio 2 conditions, the convergence behavior of the optimization algorithms is illustrated in Figure 8, which shows the accuracy rate evolution over 30 iterations for both HO-LSSVM and LCM-HO-LSSVM. The results demonstrate that LCM-HO-LSSVM achieves higher accuracy and a more stable convergence performance, with the chaotic mapping strategy effectively enhancing the optimization process. The experimental results are presented in Figure 9 and statistical results in Table 10 and Figure 10.

**Table 9:** Partial characterization data set.

1	2	3	4	5	6	7	8	…	21
0.7214	0.6775	0.4502	0.1728	0.6954	0.5994	0.5054	0.3623	…	0.7424
0.4291	0.3703	0.4522	0.3805	0.8489	0.7715	0.4140	0.5551	…	0.2023
0.6410	0.5867	0.3585	0.1887	0.7056	0.5797	0.2781	0.2988	…	0.5399
0.4126	0.3572	0.6395	0.4223	0.9915	0.9850	0.4101	0.8188	…	0.0993
0.3847	0.3312	0.2568	0.4199	0.6238	0.5513	0.2947	0.3229	…	0.2437
0.3947	0.3413	0.2598	0.3370	0.6045	0.5262	0.1845	0.3206	…	0.2955

**Table 10:** Accuracy of each classifier.

Ratio (Training:Testing)	LSSVM	HO-LSSVM	LCM-HO-LSSVM
Ratio 1 (60:20)	78.610%	98.889%	98.889%
Ratio 2 (55:25)	79.110%	98.670%	99.110%
Ratio 3 (50:30)	77.410%	98.333%	98.703%
Ratio 4 (40:40)	50.280%	88.472%	91.111%
Ratio 5 (30:50)	45.670%	79.778%	81.333%

Validation based on a multi-case complex damage-bearing dataset indicates that the LCM-HO-LSSVM model consistently outperforms the comparison algorithms across different testing scales. The experimental data reveal the following:

1. When the training set is sufficiently large, both LCM-HO-LSSVM and HO-LSSVM achieve an accuracy rate of over 98%, thereby confirming the inherent advantages of the HO framework; and when the number of training samples is N = 55, the accuracy rate of LCM-HO-LSSVM reaches 99%.
2. As the number of training samples gradually decreases to N = 55 and N = 50, the accuracy of LCM-HO-LSSVM remained stable at 98.85 ± 0.25%, consistently outperforming HO-LSSVM and LSSVM.
3. When the training samples are further reduced to smaller sizes of N = 40 and N = 30, LCM-HO-LSSVM exhibits improvements of 40.831% and 35.663%, respectively, compared to the unoptimized model.

This performance advantage arises from the synergy between multi-domain feature engineering and chaos optimization: When ample samples are available, the search capability of the HO algorithm is fully utilized; conversely, when samples are limited, the perturbation mechanism of logistic chaos mapping effectively mitigates overfitting, allowing the model to maintain stable classification performance despite a lower sample count and a feature dimension of 21D.

**Figure 11:**
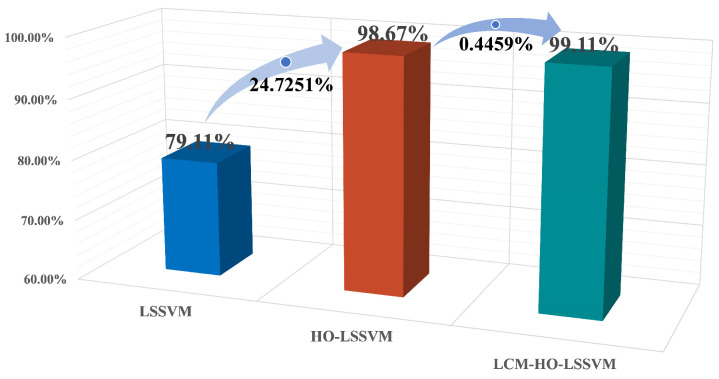
Accuracy of three classifiers in ratio 2 with different classifiers enhancement.

The experimental data indicate that LCM-HO-LSSVM demonstrates considerable advantages in the validation scenario with a sample ratio of 2:

1. The peak accuracy of the unoptimized LSSVM is 79.11%, the highest of the five scales, when the test is trained at ratio 2. In contrast, the optimized HO-LSSVM achieves an accuracy of 98.67% and the LCM-HO-LSSVM further improves to 99.11%.
2. Compared to the original LSSVM, LCM-HO-LSSVM improves by 20 percentage points in absolute terms. The accuracy of HO-LSSVM is relatively improved by 24.7251% compared to LSSVM, while the accuracy of LCM-HO-LSSVM is further improved by 0.4459% relatively compared to HO-LSSVM. These enhancements are illustrated in Figure 11.

**Figure 12:**
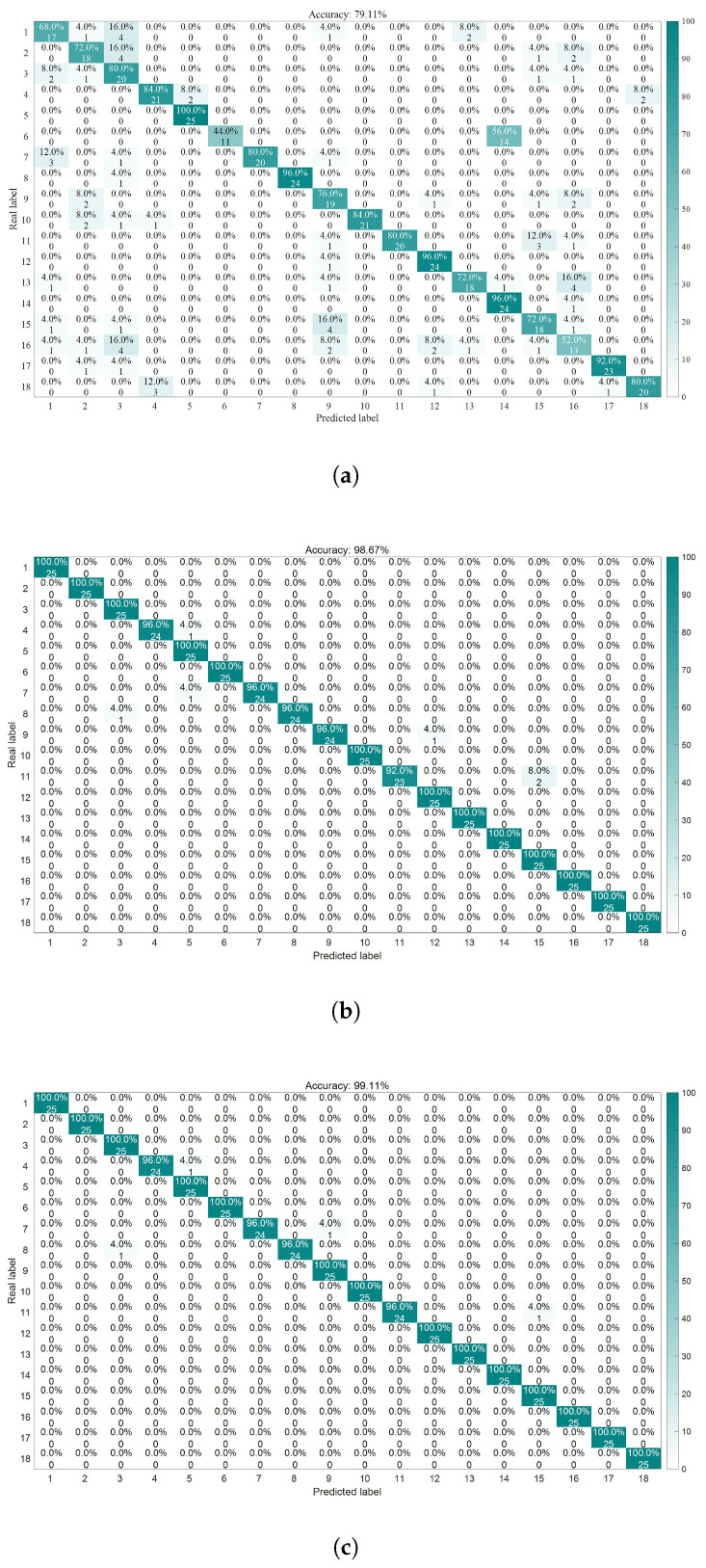
Confusion matrix diagram for three classifiers. (**a**) LSSVM confusion matrix. (**b**) HO-LSSVM confusion matrix. (**c**) LCM-HO-LSSVM confusion matrix.

As demonstrated by the confusion matrix and accuracy rates depicted in Figure 12, the findings can be summarized as follows:

1. The average accuracy of the LSSVM for healthy bearings is 80.8%, with label 1 achieving only 68% accuracy. The average accuracy for outer-ring damage is 76%, where label 6 has an accuracy of merely 44%. For inner-ring damage, the average accuracy is 83.2%, with labels 13 and 16 both achieving 72% accuracy. The average accuracy for composite damage is 74.67%, with label 16 exhibiting only 52% accuracy.
2. The average accuracy rate for the HO-LSSVM for healthy bearings is 99.2%. The average accuracy for outer ring damage is 97.6%, while for inner ring damage, it is 98.4%. The average accuracy for composite damage reaches 100%.
3. The average accuracy rate for the LCM-HO-LSSVM for healthy bearings is also 99.2%. The average accuracy for outer ring damage is 98.4%, and for inner ring damage, it is 99.2%. The average accuracy for composite damage is, agai, 100%.

In conclusion, the LCM-HO-LSSVM demonstrates a significant improvement over LSSVM across all four categories. Additionally, when compared to HO-LSSVM, there is an enhancement of 0.8% in accuracy for both outer and inner ring damage, indicating excellent generalization and robust resistance to interference in complex damage scenarios.

#### 5.2.2. Validation of the Effectiveness of Multi-Domain Feature Sets

This section also performs feature selection comparison tests to confirm the requirement and validity of the retrieved 21-dimensional multi-domain feature collection. The benchmark condition is chosen to be the highest recognition accuracy ratio of 2 (training set:test set = 55:25). Reduced-dimension feature subsets are created using various feature selection techniques, and their diagnostic performance is subsequently contrasted and examined with that of the original 21-dimensional feature set.

(1) A feature discriminative ability assessment approach based on Euclidean distance is used to eliminate feature redundancy and filter out features with the best discriminative ability from multi-dimensional features. This approach computes each feature’s separability over various fault categories in order to evaluate its discriminative power. For the *i*-th feature, the formula for calculating its discriminative ability d(i) is Equation (11).

(11) $$d\left(i\right)=\frac{\sum_{c=1}^{N_{c}}\sum_{j\neq c}\sum_{k=1}^{N_{c}}{\left(x_{ck}^{\left(i\right)}-\mu_{j}^{\left(i\right)}\right)}^{2}}{N_{c}\times N_{total}}$$

where $x_{ck}^{\left(i\right)}$ is the *i*-th feature value of the *k*-th sample of the *c*-th class. $\mu_{j}^{\left(i\right)}$ is the *i*-th feature mean of the *j*-th class. $N_{c}$ is the number of categories and $N_{total}$ is the total sample size. c=1,…4;i=1,2,…21;j=1,2,…4.

Normalization processing:

(12) $$d_{norm}\left(i\right)=\frac{d(i)}{\max_{k}d\left(k\right)}$$

where $d_{norm}\left(i\right)$ is the standardized discriminant ability value and *k* represents the index of all features.

**Figure 13:**
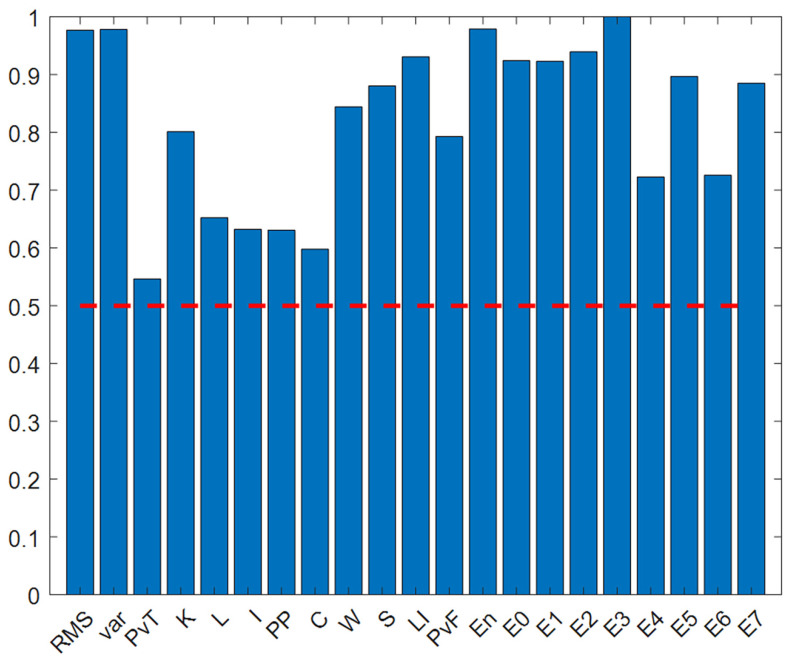
Numerical graph of European distance normalized feature discrimination.The red dotted line represents the threshold value of 0.5.

The results in Figure 13 show that the Euclidean distance discrimination values of all 21 features are greater than 0.5, indicating that these features have good discrimination capabilities. To further analyze feature redundancy, Euclidean distance thresholds of 0.6, 0.7, 0.8, and 0.9 were set to construct four feature subsets. The values obtained for each feature are shown in Table 11.

(2) The out-of-bag (OOB) importance scores for each feature were calculated using the random forest algorithm. To facilitate comparison and analysis, the original importance scores were normalized to ensure that all feature importance values were normalized to the [0, 1] interval. The normalization formula is given by Equation (13):

(13) $$I_{norm}\left(i\right)=\frac{I(i)}{\max_{j}I\left(j\right)}$$

where $I_{norm}\left(i\right)$ is the normalized importance of the *i*-th feature. I(i) is the *i*-th original OOB importance score and $\max_{j}I\left(j\right)$ is the maximum importance value among all features.

**Figure 14:**
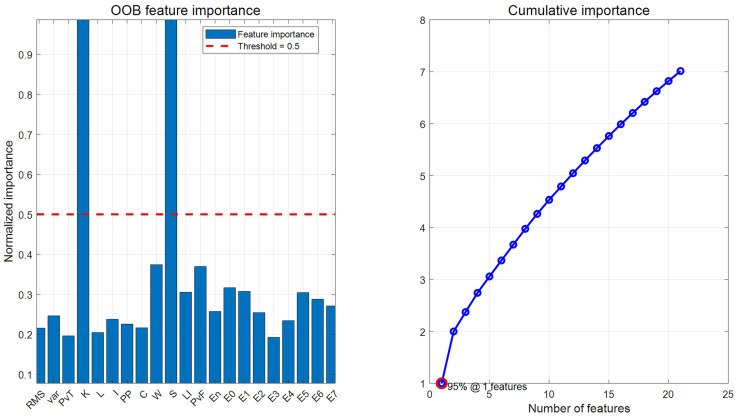
OOB feature importance and cumulative importance of each feature.

The OOB importance score results are shown in Figure 14 and Table 12. Based on the results, a TOP-K strategy was considered: the TOP-8, TOP-10, and TOP-12 feature subsets were selected based on importance rankings. Additionally, due to the uneven distribution of OOB features, a hybrid strategy was designed: By selecting the top 2 most important features, and then selecting 1–2 representative features from each of the time domain, frequency domain, and time-frequency domain, a 7-dimensional hybrid feature set was designed.

**Figure 15:**
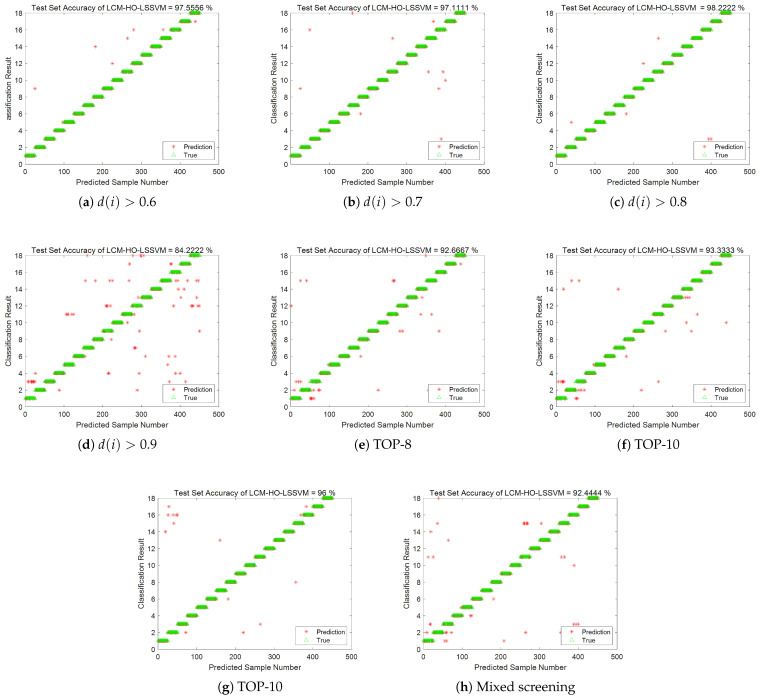
Accuracy rate of each screening scheme.

(3) The feature subsets generated by the above methods were input into the LCM-HO-LSSVM model for fault diagnosis. The accuracy is shown in Figure 15, and the results are summarized in Table 13.

Table 13 shows the following:

(1) As the Euclidean distance threshold increases, the number of features decreases, and diagnostic accuracy declines to varying degrees. When the threshold is 0.9, the decline in diagnostic accuracy is most pronounced, decreasing by 14.89% compared to the original 21-dimensional feature set.
(2) Feature selection based on random forest importance also results in accuracy loss. The diagnostic accuracies of the TOP-8, TOP-10, and TOP-12 feature sets are 92.66%, 93.33%, and 96%, respectively, all of which are lower than the 99.11% of the original feature set.
(3) Although the hybrid strategy feature set considers the balance of multi-domain features, the 7-dimensional features still cannot fully represent complex fault information, resulting in a diagnostic accuracy of 92.44%.

Through validation experiments using various mainstream feature selection methods, it was found that whether based on Euclidean distance similarity screening or random forest importance ranking selection, the diagnostic accuracy of the reduced-dimension feature subset decreased. This result confirms the effectiveness of the original 21-dimensional multi-domain feature set and the irreplaceability of each feature dimension.

### 5.3. Experiment 2—Self-Constructed Marine Bearing Test Platform

Addressing the unique characteristics of multi-bearing coupled vibrations in ship propulsion systems, this study overcomes the limitations of traditional single-bearing diagnostic methods by tackling the technical challenges of identifying rolling bearing failures in multi-bearing systems. Theoretical analysis indicates that in multi-bearing systems composed of rolling bearings and journal bearings, when a rolling bearing fails, its vibration signals not only contain the bearing’s own fault characteristics but are also influenced by coupling effects from other bearings in the system and the shaft structure, leading to nonlinear coupling phenomena such as modal mixing and transmission path modulation. These factors significantly reduce the average identification rate of traditional single-fault diagnosis methods.

To address this challenge, this study developed an experimental platform for simulating multi-bearing faults in ship propulsion shaft systems, which is innovative in several aspects:

1. Realistic operating conditions: reproduces typical variable-speed operating conditions in ship propulsion systems.
2. Flexible fault configuration: supports various combinations of inner ring, outer ring, rolling element, and composite faults in rolling bearings.
3. Signal integrity: enables synchronous acquisition of multi-directional vibration signals.

**Figure 16:**
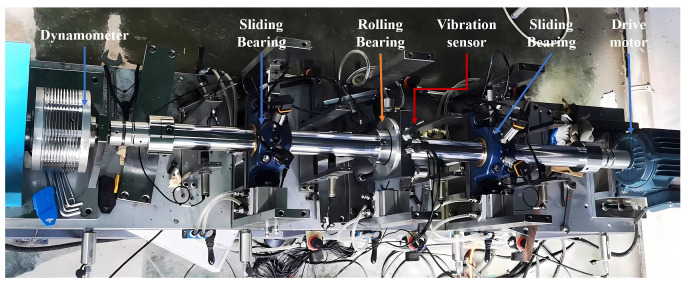
Schematic diagram of self-built platform.

The experimental system is shown in Figure 16 and consists of a three-phase asynchronous motor (1200 r/min), shaft, rolling bearings, sliding bearings, a dynamometer assembly, and several sensor assemblies. The rolling bearing model is 6214-ZZ, with specific structural parameters listed in Table 14. The sensor model is DH105E, with a response frequency of 0.1 Hz to 1000 Hz, and the signal sampling device is DH5960.

The rolling bearing fault settings are as follows: Inner and outer ring faults are created using wire cutting electrical discharge machining (EDM), with dimensions of 2.5 mm depth × 0.5 mm width. Rolling element faults are created using EDM, with a depth of 0.3 mm. Composite faults are defined as combinations of these single fault modes.

Data acquisition setup: Radial vibration acceleration signals were collected from the rolling bearings on the test bench. The monitoring was performed at a sampling rate of 20 kHz, with each sample lasting 2 s.

The dataset includes five signal categories: normal (N), rolling element failure (B), inner ring failure (IR), outer ring failure (OR) and composite failure (C). By analyzing the collected vibration signals, it was demonstrated that the improved HO-LSSVM method has significant advantages under complex ship operating conditions.

This experiment introduces three significant improvements compared to the German Paderborn University (PU) bearing dataset:

1. Expansion of Fault Types: New types of rolling element fault have been introduced and four different damage modes (B/IR/OR/C + N) have been constructed.
2. Optimization of working condition design: A single working condition test with a fixed rotational speed of 1200 r/min is employed to evaluate the robustness of the method under stable working conditions.
3. Data preprocessing: The sliding window is set to w = 1600 for the original signal, extracting s = 4000 valid fault points per sample and m = 100 standardized sample sizes for each fault type.

Table 15 presents the complete experimental data parameter system, which establishes a comprehensive method validation framework through complementary validation with the PU dataset across multiple working conditions.

#### 5.3.1. Fault Identification Results 2

**Figure 17:**
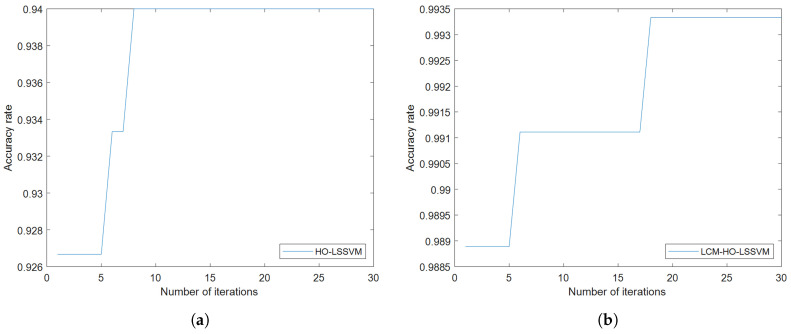
Convergence curves of HO-LSSVM and LCM-HO-LSSVM after 30 iteration. (**a**) HOLSSVM iteration curve; (**b**) LCM-HO-LSSVM iteration curve.

**Figure 18:**
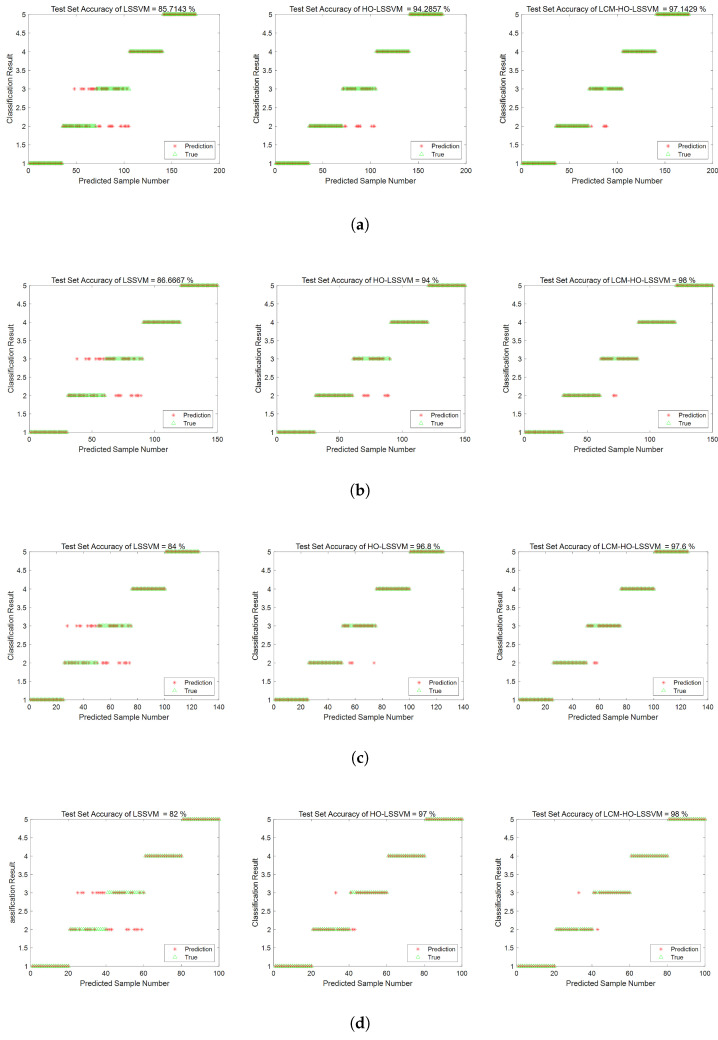
Accuracy rate of three classifiers at different ratios. (**a**) Ratio 1. (**b**) Ratio 2. (**c**) Ratio 3. (**d**) Ratio 4.

The same configuration as in Section 5.2.1 was used for the calculations. First, the multi-domain features of the signals collected by the self-built test rig were computed, resulting in a multi-domain feature dataset, with a portion of the dataset shown in Table 16. These obtained feature datasets were then, respectively, imported into the LSSVM, HO-LSSVM, and LCM-HO-LSSVM classifiers. The parameter configurations for HO and LCM-HO were consistent with those in the PU experiment. The convergence curves of the HO-LSSVM and LCM-HO-LSSVM after 30 iterations are shown in Figure 17. To evaluate the performance of the classifiers, experiments were conducted with different ratios of training and testing samples. The results are shown in Figure 18, with statistical results presented in Table 17.

**Figure 19:**
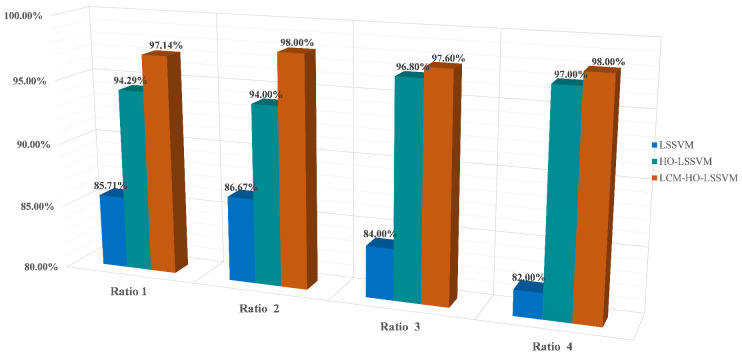
Different proportions of accuracy for different classifiers.

**Figure 20:**
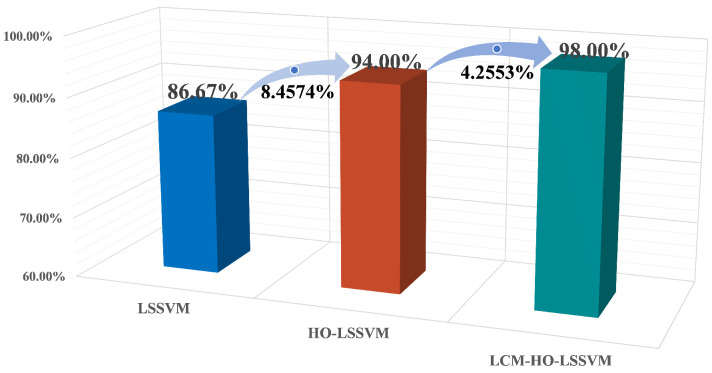
Accuracy improvement ratio of the three classifiers at ratio 2.

**Figure 21:**
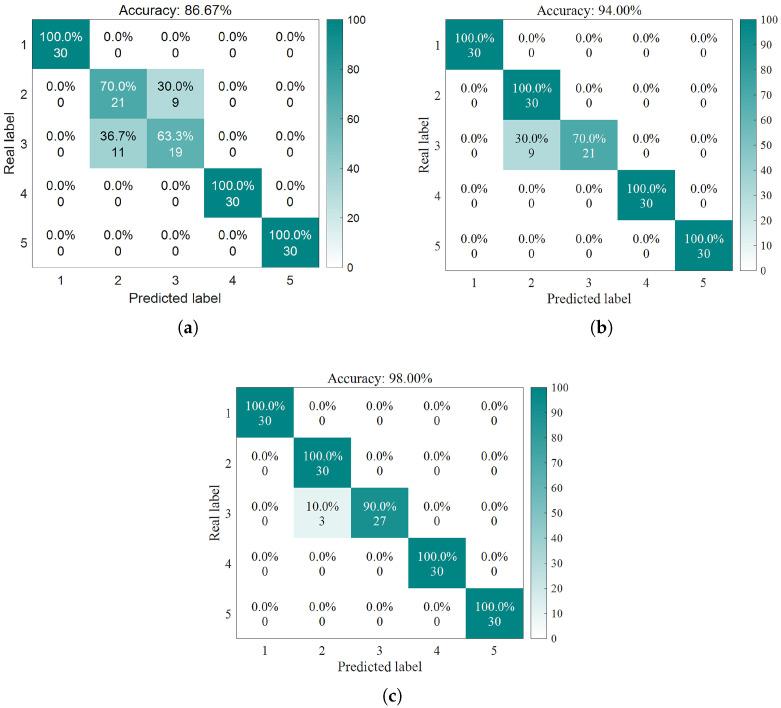
Confusion matrix diagram for three classifiers. (**a**) LSSVM confusion matrix. (**b**) HO-LSSVM confusion matrix. (**c**) LCM-HO-LSSVM confusion matrix.

Validation conducted on a specialized experimental platform for marine propulsion shaft systems demonstrates that the LCM-HO-LSSVM model exhibits superior performance in single-case multi-fault scenarios. The experimental design incorporates four types of typical bearing faults (IR, OR, B, and C) and evaluates four different training ratios. The results are summarized as follows:

1. Figure 19 illustrates the comparative results of the algorithms across varying training set ratios. The accuracy of LCM-HO-LSSVM consistently surpasses that of both the HO-LSSVM and LSSVM across all four ratios, indicating that the proposed method possesses commendable stability, generalization, and enhanced recognition capability.
2. The maximum accuracy achieved by the original LSSVM is 86.67 % (at ratio 2), while the HO-LSSVM improves this to 94.0%, and the LCM-HO-LSSVM reaches an impressive 98.0%. The improvements in accuracy are 8.4574% for the HO-LSSVM, 4.2553% for the LCM-HO-LSSVM, and a significant 13.0726% for the LCM-HO-LSSVM compared to the original LSSVM accuracy, as depicted in Figure 20.
3. The confusion matrix and accuracy metrics for ratio 2 are presented in Figure 21, respectively. The identification accuracy for the normal state (N) is 100% across all three methods. For rolling body failure (B), the LSSVM achieves only 70%, whereas both the HO-LSSVM and LCM-HO-LSSVM attain 100%, resulting in an absolute improvement of 30%. Regarding composite failure (C), the LSSVM records the lowest identification accuracy at 63.3%, while the HO-LSSVM and LCM-HO-LSSVM achieve 70% and 90%, respectively, yielding absolute improvements of 26.7% over the LSSVM and 20% over the HO-LSSVM.

The experimental data substantiate that the proposed LCM-HO-LSSVM method possesses three significant advantages. (1) Method stability: It demonstrates high consistency of results across different training ratios, exhibiting minimal fluctuations in accuracy (with a standard deviation of only 0.35%), thereby reflecting the robustness of the algorithm. (2) Multi-fault recognition capability: It exhibits excellent differentiation for various fault types, including the inner ring, outer ring, rolling body, and composite faults. (3) Engineering adaptability: The method maintains a stable and high recognition rate (>97%) even under conditions of high noise and substantial interference in the ship propulsion system, particularly for the challenging composite faults.

To further demonstrate the superiority of the proposed method, we conducted a comparative study with members of the same research group using the same experimental platform [43]. This study employed a method combining COA-VMD signal processing, GCMMPFE feature extraction, and SVM for fault recognition. However, that method only addressed four fault types (healthy, inner ring, outer ring, and rolling body faults), failing to recognize composite faults, and achieved a maximum diagnostic accuracy of 97.5%. In contrast, the model proposed in this study, while handling more complex fault types (including composite faults), achieved a higher accuracy of 98.0%. Although the accuracy improvement is 0.5%, it is noteworthy that our method’s diagnostic process is more concise and efficient. By directly performing multi-domain feature extraction on the raw signals to complete fault recognition, it eliminates cumbersome signal processing steps, making it more suitable for practical engineering applications. This comparison fully validates that our method, under similar experimental conditions, not only offers more comprehensive fault recognition capabilities and higher diagnostic precision but also holds a more significant advantage in terms of engineering applicability.

#### 5.3.2. Validation of the Effectiveness of Multi-Domain Feature Sets

Similarly, the validity and necessity of the extracted 21-dimensional multi-domain feature set in the ship data set were verified, and feature selection comparison experiments were also conducted. The highest recognition accuracy ratio of 2 (training set:test set = 70:30) was selected as the benchmark condition, and multiple feature selection strategies were used to construct reduced-dimension feature subsets, which were then compared and analyzed with the diagnostic performance of the original 21-dimensional feature set.

**Figure 22:**
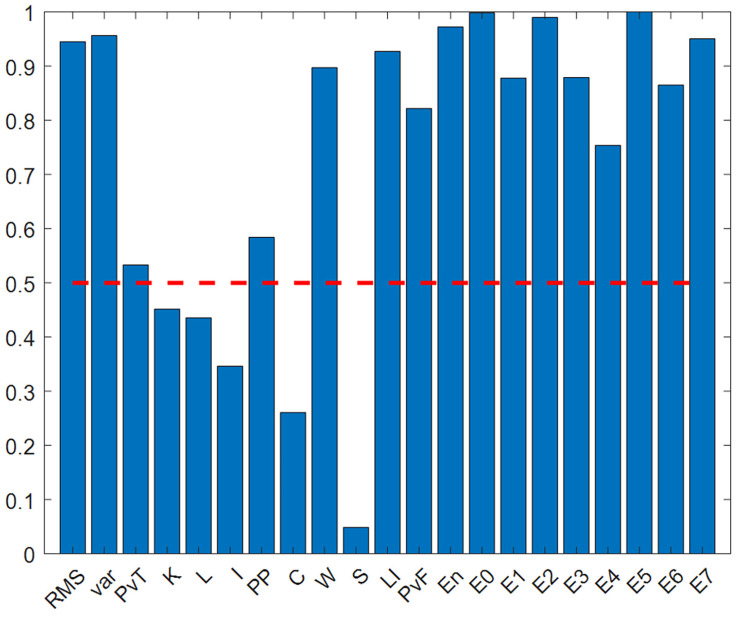
Numerical graph of European distance normalized feature discrimination.

(1) The same European distance is used, and the formula principle is the same as in Section 5.2.2. Figure 22 and Table 18 show the normalized feature discrimination ability values for all features.

From the above chart, it can be seen that the Euclidean distance discrimination values are evenly distributed, with most greater than 0.5. There are 16 features greater than 0.5 and 16 features greater than 0.6, so the threshold strategies adopted are 0.5, 0.7, and 0.9.

**Figure 23:**
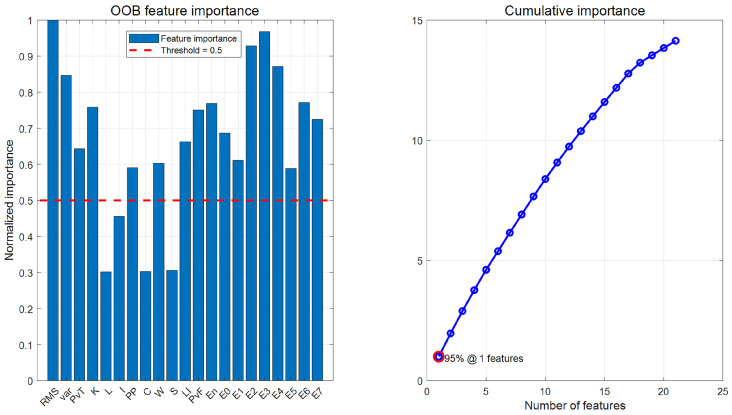
OOB feature importance and cumulative importance of each feature.

**Figure 24:**
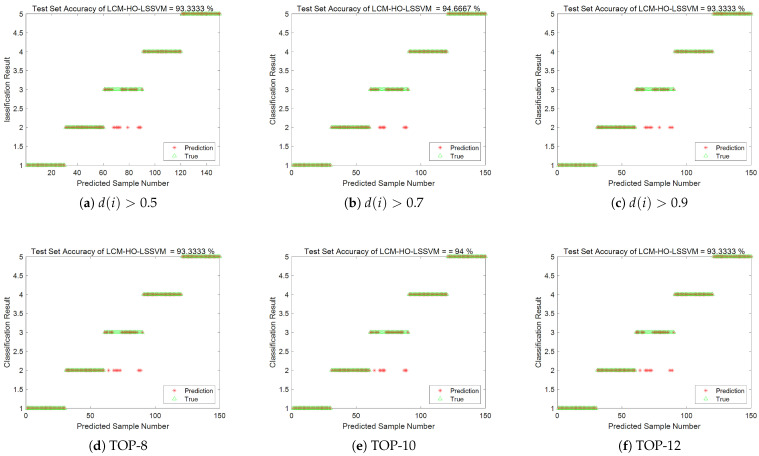
Accuracy rate of each screening scheme.

(2) Similarly, the OOB importance scores calculated using the random forest algorithm are shown in Figure 23. From the figure, it can be seen that the OOB importance distribution is uniform, so the TOP-K strategy is directly adopted, and the TOP-8, TOP-10, and TOP-12 importance scores are selected as three screening methods to generate a new feature dataset. Table 19 shows the normalized feature discrimination ability values of all features.

(3) The feature datasets obtained from the above screening method and without screening were imported into the LCM-HO-LSSVM-optimized classifier, using a training mode with a ratio of 2. The fault classification accuracy is shown in Figure 24 and summarized in Table 20:

In summary, the fault identification accuracy obtained after screening the Euclidean distance using three thresholds was lower than that of the original 21-dimensional feature set. Additionally, feature selection based on random forest importance also resulted in a loss of accuracy. The diagnostic accuracy of the feature datasets generated by the TOP-8, TOP-10, and TOP-12 strategies was approximately 93.33%, which is lower than the 99.11% accuracy of the original feature set. This result further confirms the effectiveness of the original 21-dimensional multi-domain feature set and the irreplaceability of each feature dimension.

## 6. Conclusions

This paper proposes an intelligent fault diagnosis method based on multi-domain feature extraction and the LCM-HO-LSSVM for marine bearing fault identification. The method demonstrates significant advantages:

(1) Multi-domain features are extracted directly from raw vibration signals without complex preprocessing, enhancing practical applicability for field deployment while effectively capturing bearing characteristics under various operating conditions.
(2) The LCM-HO algorithm achieves automatic LSSVM parameter tuning with enhanced population diversity, effectively avoiding local optima convergence issues.
(3) Experimental validation shows substantial improvements—99.11% accuracy on the Paderborn dataset (20.00% improvement over standard LSSVM) and 98.00% accuracy on the self-constructed platform (11.33% improvement).

The proposed method provides an effective solution for marine bearing fault diagnosis with superior accuracy, computational efficiency, and practical deployment potential for intelligent ship maintenance systems.

While the proposed method provides a solid foundation, its further enhancement and comprehensive validation constitute a promising research roadmap. Future work will, therefore, focus on the following:

(1) Practical Implementation and Computational Efficiency: We will address resource constraints and variable operating conditions by developing lightweight models and adaptive signal processing strategies. The method’s runtime statistics and computational efficiency will be analyzed through hardware benchmarking to ensure its suitability for near-real-time industrial applications.
(2) Model Enhancement and Advanced Benchmarking: We will benchmark our approach against mainstream deep learning models (such as CNNs and Transformers) and explore advanced multi-sensor fusion strategies to further enhance diagnostic accuracy and robustness.
(3) Statistical Validation and Experimental Rigor: We will enhance the statistical rigor of our validation by adopting k-fold cross-validation and applying statistical significance tests to confirm the reliability of our observed performance gains.
(4) Dataset Expansion: Expanding dataset diversity across broader bearing types and marine environments remains a priority to further improve diagnostic accuracy and robustness.

## Figures and Tables

**Table 1:** Time-domain feature.

Feature Name	Formula	Feature Name	Formula
RMS	$RMS=\sqrt{\frac{1}{n}\sum_{i=1}^{n}x_{i}^{2}}$	I	$I=\frac{PvT}{\frac{1}{n}\sum_{i=1}^{n}\left|x_{i}\right|}$
Var	$\mathrm{var}=\frac{1}{n}\sum_{i=1}^{n}{\left(x_{i}-\bar{x}\right)}^{2}$	PP	$PP=\max\left(x_{i}\right)-\min\left(x_{i}\right)$
PvT	$PvT=\max\left(\left|x_{i}\right|\right)$	C	$C=\frac{PvT}{RMS}$
K	$K=\frac{\sum_{i=1}^{n}{\left(x_{i}-\bar{x}\right)}^{4}}{n\times {\mathrm{var}}^{2}}$	W	$W=\frac{RMS}{\frac{1}{n}\sum_{i=1}^{n}\left|x_{i}\right|}$
L	$L=\frac{PvT}{{\left(\frac{1}{n}\sum_{i=1}^{n}\sqrt{\left|x_{i}\right|}\right)}^{2}}$	S	$S=\frac{\frac{1}{n}\sum_{i=1}^{n}{\left(x_{i}-\bar{x}\right)}^{3}}{{\left(\sqrt{\frac{1}{n}\sum_{i=1}^{n}{\left(x_{i}-\bar{x}\right)}^{2}}\right)}^{3}}$
LI	$LI=\sum_{i=0}^{n}\left|x_{i+1}-x_{i}\right|$		

**Table 2:** Frequency domain, time-frequency features.

Feature Name	Formula	Feature Name	Formula
PvF	$PvF=\max\left(r_{k}\right)$	wavelet packet decomposition	$E_{j}=\sum_{i=1}^{N}x_{ji}^{2}$
En	$En=\sum_{f=1}^{N}r_{f}^{2}$		

**Table 3:** Test function list.

Fn	Formula	Range	$f_{\min}$
F1	$F_{1}=\sum_{i=1}^{n}x_{i}^{2}$	[−100,100]	0
F2	$F_{2}=\sum_{i=1}^{n}\left|x_{i}\right|+\prod_{i=1}^{n}\left|x_{i}\right|$	[−10,10]	0
F3	$F_{3}=\max\left\{\left|x_{i}\right|,1\leq i\leq n\right\}$	[−100,100]	0
F4	$F_{4}=\sum_{i=1}^{n}ix_{i}^{4}+random\left[0,1\right]$	[−1.28,1.28]	0
F5	$F_{5}=-20\exp\left(0.2\sqrt{\frac{1}{n}\sum_{i=1}^{n}x_{i}^{2}}\right)-\exp\left(\frac{1}{n}\sum_{i=1}^{n}\cos\left(2\pi x_{i}\right)\right)+20+e$	[−32,32]	0
F6	$F_{6}=\frac{1}{4000}\sum_{i=1}^{n}x_{i}^{2}-\prod_{i=1}^{n}\cos\left(\frac{x_{i}}{\sqrt{i}}\right)+1$	[−600,600]	0
F7	$F_{7}=\sum_{i=1}^{n}x_{i}^{2}+{\left(\sum_{i=1}^{n}0.5ix_{i}\right)}^{2}+{\left(\sum_{i=1}^{n}0.5ix_{i}\right)}^{4}$	[−5,10]	0

**Table 4:** Function test results.

Fn	Indicators	LCM-HO	HO	SSA	GWO
F1	Min	0	0	0	$9.19\times 10^{-23}$
	Avg	$2.65\times 10^{-290}$	$1.61\times 10^{-287}$	$4.09\times 10^{-47}$	$1.88\times 10^{-21}$
	Std	0	0	$2.24\times 10^{-46}$	$1.71\times 10^{-21}$
	Max	$6.15\times 10^{-289}$	$4.42\times 10^{-286}$	$1.23\times 10^{-45}$	$7.18\times 10^{-21}$
F2	Min	$1.64\times 10^{-159}$	$2.41\times 10^{-158}$	$5.20\times 10^{-137}$	$9.73\times 10^{-14}$
	Avg	$2.77\times 10^{-146}$	$6.09\times 10^{-145}$	$6.98\times 10^{-26}$	$4.39\times 10^{-13}$
	Std	$1.21\times 10^{-145}$	$2.67\times 10^{-144}$	$3.28\times 10^{-25}$	$2.44\times 10^{-13}$
	Max	$6.60\times 10^{-145}$	$1.42\times 10^{-143}$	$1.78\times 10^{-24}$	$1.31\times 10^{-12}$
F3	Min	$1.68\times 10^{-158}$	$7.37\times 10^{-156}$	$1.24\times 10^{-111}$	$4.25\times 10^{-6}$
	Avg	$2.97\times 10^{-147}$	$6.46\times 10^{-145}$	$8.33\times 10^{-28}$	$2.94\times 10^{-5}$
	Std	$9.89\times 10^{-147}$	$2.35\times 10^{-144}$	$3.81\times 10^{-27}$	$4.24\times 10^{-5}$
	Max	$5.21\times 10^{-146}$	$1.21\times 10^{-143}$	$2.07\times 10^{-26}$	$2.25\times 10^{-4}$
F4	Min	$4.79\times 10^{-6}$	$1.58\times 10^{-6}$	$9.12\times 10^{-5}$	$4.34\times 10^{-4}$
	Avg	$1.84\times 10^{-4}$	$2.31\times 10^{-4}$	$2.60\times 10^{-3}$	$2.94\times 10^{-3}$
	Std	$1.50\times 10^{-4}$	$1.74\times 10^{-4}$	$2.96\times 10^{-3}$	$1.30\times 10^{-3}$
	Max	$5.77\times 10^{-4}$	$6.02\times 10^{-4}$	$1.19\times 10^{-2}$	$5.86\times 10^{-3}$
F5	Min	$4.44\times 10^{-16}$	$4.44\times 10^{-16}$	$4.44\times 10^{-16}$	$7.51\times 10^{-14}$
	Avg	$4.44\times 10^{-16}$	$4.44\times 10^{-16}$	$4.44\times 10^{-16}$	$9.27\times 10^{-14}$
	Std	0	0	0	$1.23\times 10^{-14}$
	Max	$4.44\times 10^{-16}$	$4.44\times 10^{-16}$	$4.44\times 10^{-16}$	$1.21\times 10^{-13}$
F6	Min	0	0	0	0
	Avg	0	0	0	$4.92\times 10^{-3}$
	Std	0	0	0	$1.01\times 10^{-2}$
	Max	0	0	0	$3.27\times 10^{-2}$
F7	Min	0	0	$1.82\times 10^{-263}$	$3.04\times 10^{-2}$
	Avg	$1.00\times 10^{-289}$	$2.07\times 10^{-286}$	$1.02\times 10^{-10}$	$9.18\times 10^{-1}$
	Std	0	0	$4.84\times 10^{-10}$	$1.44\times 10^{0}$
	Max	$2.85\times 10^{-288}$	$6.21\times 10^{-285}$	$2.65\times 10^{-9}$	$5.64\times 10^{0}$

**Table 11:** Euclidean distance feature discrimination normalized value.

1	2	3	4	5	6	7	8	9	10	
0.977	0.978	0.546	0.801	0.652	0.632	0.631	0.598	0.844	0.880	
11	12	13	14	15	16	17	18	19	20	21
0.931	0.793	0.980	0.924	0.923	0.939	1	0.723	0.897	0.726	0.885

**Table 12:** All features’ OOB importance and their normalized values.

	**RMS**	**Var**	**PvT**	**K**	**L**	**I**	**PP**	**C**	**W**	**S**
Importance	0.861	0.984	0.783	3.984	0.816	0.949	0.901	0.864	1.493	3.993
Normalization	0.216	0.246	0.196	0.998	0.204	0.238	0.226	0.216	0.374	1
**LI**	**PvF**	**En**	**E0**	**E1**	**E2**	**E3**	**E4**	**E5**	**E6**	**E7**
1.219	1.476	1.027	1.263	1.228	1.015	0.768	0.936	1.216	1.149	1.082
0.305	0.370	0.257	0.316	0.307	0.254	0.192	0.234	0.305	0.288	0.271

**Table 13:** Recognition accuracy of LCM-HO-LSSVM using different methods.

Screening Strategy		Number of Selected Features	LCM-HO-LSSVM Accuracy
European distance	d(i)>0.6	19	97.55%
	d(i)>0.7	16	97.11%
	d(i)>0.8	13	98.22%
	d(i)>0.9	8	84.22%
OOB importance TOP-K	TOP-8	8	92.66%
	TOP-10	10	93.33%
	TOP-12	12	96%
	Mixed screening	7	92.44%
Unscreened		21	99.11%

**Table 14:** Experimental rolling bearing structural parameters.

6214-ZZ	Parameter Name	Number of Parameters
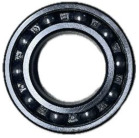	Inner diameter	70 mm
	Outer diameter	125 mm
	Width	24 mm
	Number of balls	10
	static load	45 kN
	dynamic load	60.8 kN
	Maximum speed (grease lubrication)	4800 r/min
	Maximum speed (oil lubrication)	6000 r/min

**Table 15:** Signal data label classification.

Bearing Condition Type	Sample Size	Label
Normal (N)	100	1
Rolling body failure (B)	100	2
Composite failure (C)	100	3
Inner ring failure IR	100	4
Outer ring failure (OR)	100	5

**Table 16:** Partial characterization dataset.

1	2	3	4	5	6	7	8	…	21
0.1133	0.0387	0.1434	0.0634	0.5902	0.6022	0.1679	0.5962	…	0.0344
0.1098	0.0371	0.1618	0.0680	0.6774	0.7115	0.1786	0.7367	…	0.0291
0.1123	0.0382	0.1618	0.0644	0.6742	0.7016	0.1786	0.7186	…	0.0329
0.1111	0.0376	0.1618	0.0680	0.6925	0.7207	0.1564	0.7274	…	0.0374
0.1064	0.0356	0.1276	0.0735	0.5745	0.5741	0.1118	0.5396	…	0.0341
0.1066	0.0357	0.1460	0.0780	0.6664	0.6833	0.1632	0.6575	…	0.0289

**Table 17:** Accuracy of different proportions of classifiers.

Ratio (Training:Testing)	LSSVM	HO-LSSVM	LCM-HO-LSSVM
Ratio 1 (65:35)	85.7143%	94.2857%	97.1429%
Ratio 2 (70:30)	86.6667%	94%	98%
Ratio 3 (75:25)	84%	96.8%	97.6%
Ratio 4 (80:20)	82%	97%	98%

**Table 18:** Euclidean distance feature discrimination normalized value.

1	2	3	4	5	6	7	8	9	10	
0.945	0.957	0.533	0.452	0.436	0.346	0.584	0.261	0.897	0.049	
11	12	13	14	15	16	17	18	19	20	21
0.927	0.822	0.973	0.998	0.878	0.990	0.879	0.754	1	0.865	0.951

**Table 19:** All features’ OOB importance and their normalized values.

	**RMS**	**Var**	**PvT**	**K**	**L**	**I**	**PP**	**C**	**W**	**S**
Importance	0.690	0.584	0.444	0.524	0.208	0.315	0.408	0.209	0.416	0.211
Normalization	1	0.847	0.643	0.759	0.301	0.456	0.590	0.303	0.603	0.306
**LI**	**PvF**	**En**	**E0**	**E1**	**E2**	**E3**	**E4**	**E5**	**E6**	**E7**
0.457	0.518	0.531	0.474	0.422	0.641	0.668	0.601	0.406	0.533	0.500
0.662	0.751	0.769	0.687	0.611	0.929	0.968	0.871	0.588	0.772	0.725

**Table 20:** Recognition accuracy of LCM-HO-LSSVM using different methods.

Screening Strategy		Number of Selected Features	LCM-HO-LSSVM Accuracy
European distance	d(i)>0.5	16	93.33%
	d(i)>0.7	14	94.67%
	d(i)>0.9	8	93.33%
OOB importance TOP-K	TOP-8	8	93.33%
	TOP-10	10	94%
	TOP-12	12	93.33%
Unscreened		21	98%

## Data Availability

The raw data supporting the conclusions of this article will be made available by the authors upon request.
